# Contemporary Management of Recurrent Nodal Disease in Differentiated Thyroid Carcinoma

**DOI:** 10.5041/RMMJ.10233

**Published:** 2016-01-28

**Authors:** Shorook Na’ara, Moran Amit, Eran Fridman, Ziv Gil

**Affiliations:** The Head and Neck Center, Department of Otolaryngology Head and Neck Surgery, Rambam Healthcare Campus, Clinical Research Institute at Rambam, Rappaport Institute of Medicine and Research, The Technion, Israel Institute of Technology, Haifa, Israel

**Keywords:** Lymph nodes, persistent, thyroid neoplasm

## Abstract

Differentiated thyroid carcinoma (DTC) comprises over 90% of thyroid tumors and includes papillary and follicular carcinomas. Patients with DTC have an excellent prognosis, with a 10-year survival rate of over 90%. However, the risk of recurrent tumor ranges between 5% and 30% within 10 years of the initial diagnosis. Cervical lymph node disease accounts for the majority of recurrences and in most cases is detected during follow-up by ultrasound or elevated levels of serum thyroglobulin. Recurrent disease is accompanied by increased morbidity. The mainstay of treatment of nodal recurrence is surgical management. We provide an overview of the literature addressing surgical management of recurrent or persistent lymph node disease in patients with DTC.

## INTRODUCTION

Differentiated thyroid cancer (DTC), which includes papillary and follicular cancer, accounts for the vast majority of thyroid cancers.^1^ Papillary thyroid carcinoma is the most common type of DTC and comprises about 80%–85% of all follicular cell-derived thyroid cancers.^2^–^5^ Patients with DTC have excellent prognosis, with a 10-year overall survival of over 90%.^6^,^7^

Generally, the outcome of DTC is usually measured by disease-specific survival, locoregional control, and postoperative complications. Regional recurrence in the cervical lymph nodes is the result of persistent disease in most cases.^8^ Recurrence harbors increased morbidity and mortality in high-risk groups and frequently requires surgical treatment in the form of neck dissection.^9^–^12^

This article provides an overview of the literature on the management of recurrent or persistent nodal disease in patients with DTC.

## NATURAL HISTORY OF RECURRENT/PERSISTENT DTC

The treatment of primary DTC is surgical excision. Surgery often involves total thyroidectomy, with therapeutic lymph node dissection when evidence of regional spread exists.^13^ High-risk patients with locally invasive or metastatic disease are usually treated by surgery followed by radioactive iodine (RAI) and thyroid stimulating hormone (TSH) suppression using L-thyroxine.^13^ Prophylactic central lymph node dissection in primary surgery is not considered standard of care.^14^

Most patients with DTC experience no recurrence and have good prognosis after the primary treatment. Yet, according to several reports, the disease recurs in 5%–30% of patients within 10 years from the initial diagnosis, depending on the primary tumor stage and treatment modality.^3^,^6^,^15^–^18^ The most common pattern of failure is regional or cervical nodal recurrence, which is associated with an increased mortality rate of 7%.^3^,^6^ The average time to regional recurrence is 2.6 years and ranges from 2 to 5 years from diagnosis (Table 1). These relapses most probably reflect persistence of microscopic disease at the lymphatic basins that was not detected before the initial operation.^8^

**Table 1.** Demographics and Treatment Modalities Reported by the Reviewed Studies.

**Author**	**Year**	***n***	**Gender F:M**	**Mean Age (y)**	**Site of LNR**		**Mean TTR (mo)**	**Surgical Treatment**		**Post-op Treatment**	
					**Central**	**Lateral**		**CCND**	**LCND**	**I131**	**RT**
Lango et al.^19^	2013	29	22:7	42	25	22	n/a	n/a	n/a	29	3
Lang et al.^20^	2013	50	35:15	54.2	50	0	36.4	50	24	50	3
Tufano et al.^21^	2012	120	78:42	40.5	120	71	36	120	n/a	n/a	n/a
Hughes et al.^22^	2012	61	42:19	40.7	61	0	24.7	31	50	13	n/a
Shah et al.^23^	2012	82	57:25	42.6	82	41	n/a	82	46	50	n/a
Roh et al.^24^	2011	45	34:11	49.2	25	24	72	45	24	n/a	n/a
Clayman et al.^25^	2011	210	125:85	42	210	0	21	210	159	70	33
Yim et al.^26^	2011	83	59:24	42	n/a	n/a	27	n/a	79	18	n/a
Al-Saif et al.^12^	2010	60	48:12	41	n/a	n/a	36	60	57	n/a	n/a
Schuff et al.^27^	2008	74	54:20	47	74	n/a	n/a	74	64	n/a	n/a
Lee et al.^28^	2008	15	n/a	n/a	n/a	n/a	n/a	8	7	n/a	n/a
Kim et al.^29^	2004	13	n/a	49.4	13	0	n/a	13	9	n/a	n/a

CCND, central compartment neck dissection; F:M, female:male; LCND, lateral compartment neck dissection; LNR, lymph node recurrence; *n*, number of patients; n/a, not available; Post-op, postoperative; RT, radiotherapy; TTR (mo), time to recurrence (months); y, years.

## DIAGNOSTIC WORK-UP FOR NODAL RECURRENCE IN DTC

High-resolution ultrasound (US) and fine-needle aspiration-proven cytology are the mainstay modalities for the diagnosis of nodal recurrence.^15^ Serum thyroglobulin (Tg) levels are routinely monitored to evaluate remission following surgery and adjuvant RAI treatment. Thyroglobulin is secreted exclusively by follicular thyroid cells, and elevated levels after total thyroidectomy and RAI therapy may suggest tumor recurrence.^9^,^30^,^31^ Stimulation of TSH enhances Tg assay sensitivity, but the impact of this test on patient outcome is controversial.^13^,^30^ Of note is that anti-Tg antibodies are detectable in 15%–20% of patients,^13^,^32^ and their presence interferes with Tg assessment. Whole-body iodine scans can also help the detection of regional and distant metastasis.^33^

The use of CT and PET-CT in recurrent disease is less established. A recent study compared the roles of US, CT, and PET-CT in the evaluation of cervical recurrence in DTC, by correlating findings with final pathology.^34^ The sensitivity, specificity, and accuracy were 69.2%, 89.7%, and 80.0% for ultrasound; 63.5%, 94.8%, and 80.0% for CT; and 53.8%, 79.3%, and 67.3% for PET-CT, respectively. The sensitivity and specificity of ultrasound and CT were higher than for PET-CT.^34^ Still, PET-CT is a valid imaging modality for the diagnosis of iodine-negative lesions on iodine scan.^35^

## RISK FACTORS FOR NODAL RECURRENCE

Papillary thyroid cancer has a propensity for lymphoinvasion. The most established risk factor for lymph node recurrence in DTC is the presence of lymph node metastasis in the primary disease.^36^–^39^ Yet, there is still controversy whether microscopic lymph node metastasis in the primary disease holds the same risk for recurrence as does macroscopic metastasis.^40^ In a study of 545 patients with papillary thyroid cancer treated with surgery with or without RAI, the 10-year risk of lymph node recurrence was 19% in macroscopic nodal involvement and 4% in microscopic lymph node involvement.^41^ Extrathyroid extension at recurrence is associated with an increased risk of recurrence.^19^,^38^,^39^,^41^ Additional risk factors for nodal recurrence include male gender, tumor size of >4 cm, and elevated thyroglobulin levels measured 6–12 months after initial treatment.^41^

## PATTERNS OF FAILURE

Regional recurrence after total thyroidectomy most commonly involves the central neck compartment and, to a lesser extent, the lateral neck (Table 1).^6^,^42^ Lateral lymph node recurrence usually occurs in the ipsilateral side and more frequently involves levels II, III, and IV.^38^ However, when lymph node metastasis is evident in the primary disease, involvement of level V should be assessed by ultrasonography.^38^

## SURGICAL MANAGEMENT OF NODAL RECURRENCE

For persistent or recurrent nodal disease, the treatment modalities available include surgical treatment or RAI. However, in low- or intermediate-risk patients with nodes of less than 1 cm, watchful waiting is a valid option.^6^,^43^ According to the 2015 American Thyroid Association (ATA) guidelines, the recommended management of suspected structural recurrence is neck dissection for patients with biopsy-proven persistent or recurrent disease greater than 8 mm in the central neck or 10 mm in the lateral neck, in the smallest dimension.^44^ Lymph node recurrence is usually characterized by well-defined masses isolated from the surrounding structures, in the absence of extracapsular extension. Recurrence at the thyroid bed is less organized and may involve the recurrent laryngeal nerve (RLN) or the trachea.^14^

Analysis of 100 patients who underwent neck dissection for recurrent nodal disease after RAI^45^ showed that the negative predictive values of US for the specific levels were: level II, 66.2%; level III, 49.4%; level IV, 55.4%; and level V, 88.5%. Consequently, a formal lateral compartment neck dissecttion, including levels II–IV with or without level V, should be the routine practice for the management of nodal recurrence.^44^,^45^

## MANAGEMENT OF THE CENTRAL NECK IN LATERAL NODAL RECURRENCE

The primary echelons for malignant tumors of the thyroid are the paratracheal, pretracheal, and superior mediastinum lymph nodes (levels VI and VII). Palpable neck disease or radiological evidence of nodal metastases in DTC will necessitate therapeutic neck dissection; however, the rationale for prophylactic neck dissection remains debatable due to lack of level I evidence. Elective central neck dissection (CND) detects occult nodal metastases in 40%–60% of the remaining patients, whereas elective lateral neck dissection (LND) detects positive nodes in 40%.^46^ Contralateral occult paratracheal nodes are evident in 10%–25% of patients.^47^,^48^ When jugular chain metastases are present, the risk of central compartment metastases is higher.^49^ Taken together, the overall risk of occult nodal metastases in patients with DTC according to some reports may reach 80%.^46^ Based on these numbers alone, some surgeons have recommended prophylactic central neck dissection (ipsilateral or bilateral) in patients with DTC with clinically positive jugular nodes.^50^ However, no data in the literature show any clinical advantage of elective CND in this population.^51^ As well, although postoperative complications of central neck dissection are relatively infrequent in experienced hands, hypocalcemia and vocal cord paralysis can lead to significant morbidity and impaired quality of life.^52^ Existing data show that prophylactic neck dissection does not influence early outcome of patients with DTC and that the risk of locoregional recurrence and distant metastases remains similar regardless of central nodes excision.^53^ Furthermore early tumor recurrence and disease-specific survival are not influenced by elective central neck dissection in patients with positive jugular chain nodes.^54^ Given the lack of objective benefit and a potential evidence of harm, prophylactic central neck dissection cannot be routinely recommended in this population.

## POSTOPERATIVE COMPLICATIONS

Reoperation of the lateral or central neck is challenging due to scarring, fibrosis, and disruption of the normal anatomy.^10^,^24^,^55^,^56^ Table 2 summarizes the complications reported following reoperation for recurrent or persistent nodal disease in DTC.

**Table 2.** Reported Complication Rates after Reoperation for Lymph Node Recurrence.

**Author**	**Year**	**Complication Rates, *n* (%)**						
		**Transient HC**	**Permanent HC**	**Transient VC Paralysis**	**Permanent VC Paralysis**	**Chyle Leak**	**Hematoma**	Other^*^
Lang et al.^20^	2013	7 (14)	0	6 (12)	1 (2)			2 (4)
Tufano et al.^21^	2012	12 (10)	3 (2.5)	0	7 (5.8)			
Hughes et al.^22^	2012	6 (9.8)	0	0	1 (1.6)	1 (1.6)		2 (3.2)
Shah et al.^23^	2012	17 (20.7)	6 (7.3)	3 (3.6)	3 (3.6)			
Clayman et al.^25^	2011	0	2 (0.9)	0	4 (1.9)			
Schuff et al.^27^	2008	12 (15.1)	5 (6.3)	1 (1.2)	0		9 (11.3)	1 (1.2)
Kim et al.^29^	2004	3 (15)	1 (5)	0	0			

* Other complications included: seroma, esophageal injury, surgical site infection, and one intraoperative death. HC, hypocalcemia; *n*, number; VC, vocal cord.

The most common complications encountered in neck dissection for recurrent disease are vocal cord paralysis and hypoparathyroidism.^29^,^57^ Transient vocal cord paralysis occurred in 0%–12% and permanent paralysis in 0%–5.8% of patients. The incidence of permanent hypocalcemia ranges between 0% and 9%, and of transient hypocalcemia between 7% and 60%.^10^,^55^,^58^,^59^ Other reported complications included chyle leak, surgical bed hematoma, and surgical site infection.

## SURGICAL OUTCOMES

The primary outcomes following surgery include locoregional control and biochemical response. Biochemical response is defined as undetectable baseline or stimulated Tg levels in the absence of structural evidence of recurrence measured in the time period of 6–24 months following treatment.^60^ Five-year disease-free rates following reoperation reach 70%.^9^,^27^ Complete biochemical response can be accomplished in about 21% of operated patients, while decreased Tg levels might reach 98% during a mean follow-up of 15.5 months.^22^ A follow-up period of up to 60 months might be needed to monitor complete biochemical response after surgery.^12^

## THE USE OF EBRT IN RECURRENT DTC

The use of external beam radiotherapy (EBRT) in DTC is controversial, and its long-term benefits remain inconclusive. In a retrospective analysis at the Memorial Sloan–Kettering Cancer Center of patients with DTC who received EBRT for locally advanced or recurrent extensive locoregional disease, the locoregional control rates were satisfactory taking into account the patients’ originally poor prognosis. Acute adverse effects of EBRT included grade 3 acute mucositis and dysphagia, while late adverse toxicity included mainly the need for gastrostomy tube insertion in 5% of the patients.^61^

The 2015 ATA guidelines state that EBRT is to be considered in patients with non-resectable loco-regional recurrences, or in cases of extranodal extension or involvement of soft tissue. They also recommend using EBRT in patients without evidence of distant metastasis.^44^

## CONCLUSIONS

Recurrent or persistent lymph node disease is the most common pattern of recurrence in DTC. The diagnosis is based mainly on US, Tg levels, and whole-body iodine scan. Nodal recurrence is best treated surgically when a structural lesion is evident. Surgery should be performed with intent to cure and includes a formal compartment neck dissection. Reoperation can be performed with low complication rates.

## Abbreviations:

ATA: American Thyroid Association
CND: central neck dissection
DTC: differentiated thyroid carcinoma
LND: lateral neck dissection
RAI: radioactive iodine
Tg: thyroglobulin
TSH: thyroid stimulating hormone
US: ultrasound
RLN: recurrent laryngeal nerve.
